# Supplementary material for: Metabolic pathways inferred from a bacterial marker gene illuminate ecological changes across South Pacific frontal boundaries
Source: Nat Commun. 2021 Apr 13;12:2213. doi: 10.1038/s41467-021-22409-4 (PMC8044245; doi:10.1038/s41467-021-22409-4)
Supplement: Supplementary file 1 — Supplementary Information [file 41467_2021_22409_MOESM1_ESM.pdf]

Supplementary Information

Metabolic pathways inferred from a bacterial marker gene illuminate ecological changes across South Pacific frontal boundaries

Eric J. Raes[1,2*], Kristen Karsh[1], Swan L. S. Sow[1,3,4], Martin Ostrowski[5], Mark Brown[6], Jodie van de Kamp[1], Rita M. Franco-Santos[3], Levente Bodrossy[1], Anya M. Waite[2]

1) CSIRO Oceans and Atmosphere, GPO Box 1538, Hobart, TAS, 7004 Australia

2) Ocean Frontier Institute and Dept. of Oceanography, Dalhousie University, Halifax, B3H 4R2 Nova Scotia, Canada

3) Institute for Marine and Antarctic Studies, University of Tasmania, 20 Castray Esplanade, Hobart 7004, TAS, Australia

4) NIOZ Royal Netherlands Institute for Sea Research, Department of Marine Microbiology and Biogeochemistry, P.O. Box 59, 1790 AB Den Burg, The Netherlands

5) Climate Change Cluster, University of Technology Sydney, Sydney, NSW, Australia

6) School of Environmental and Life Sciences, The University of Newcastle, Callaghan, NSW, Australia

*** Corresponding author:**

Eric Jorden Raes

ejraes@gmail.com

Supplementary Methods

1. *DNA sequencing and bioinformatics*

Amplicon sequence variant (ASV) tables were prepared after Bissett, Fitzgerald [1] and as outlined in https://www.australianmicrobiome.com/protocols/16sanalysisworkflow/. Briefly, FLASH v1.2.11 [2] was used to merge paired-end reads and unique sequences were denoised into 79073 ASVs with the UNOISE algorithm [3] using USEARCH 64 bit v8.0.1517 [4]. ASV abundance profiles per sample were constructed by mapping all the reads to the unique ASVs using the USEARCH 'otutab' command. Please see the https://github.com/EricRaes/marker_gene_manuscript repository for the code and information on the clustering at the 97% similarity threshold. Genomic data are available at https://www.ncbi.nlm.nih.gov/bioproject/385736 under the accession no. PRJNA385736

2. *Shotgun Metagenomic sequencing*

Illumina Novaseq shotgun sequencing was used to complement the 16S rRNA gene data. gDNA shearing and library preparations were done at the Ramaciotti centre for Genomics (UNSW, Sydney, Australia). The sample input for the half reaction Nextera Flex library prep was between 3 and 4.5ng of DNA which underwent 12 cycles of amplification. The libraries were cleaned with the Illumina Sample Purification Beads provided with the preparation kit. The libraries were QCed using Picogreen and Labchip for quantification and qualification respectively. The Libraries were then pooled using the Janus NGS liquid handling system and the pool underwent a 0.8x ratio final clean up with the AMPure beads to remove any leftover primer dimer. The pool was sequenced on the Illumina Novaseq 6000 with the S4 kit. Loading concentration used was 290pM per pool and 1% of the bacteriophage PhiX was

spiked-in to increase the diversity of the pool and as a control. Each sample was split over 4 lanes. The lanes files from each sample were then concatenated and FastQC (version: 0.11.8; Andrews [5]) was used to visualise the quality of the bases.

3. Functional microbial community composition

The main idea of PICRUSt2 is to infer the genomic content for a given 16S rRNA sequence based on averaging over the reference genomes that are most similar in 16S rRNA sequences [6, 7, 8, 9, 10]. The PICRUSt2 genome database (as of November 8, 2017) is based on 41,926 bacterial and archaeal genomes from the Integrated Microbial Genomes (IMG) database [11]. The nearest sequenced taxon index (NSTI) score in PICRUSt2 is a metric to evaluate the degree to which microorganisms in a sample are related to available sequenced genomes. PICRUSt2 outputs MetaCyc pathway predictions (Caspi et al., 2018) which are comparable with common shotgun metagenomics output abundances. MetaCyc pathways were linked to their respective secondary superclass levels in the MetaCyc pathway hierarchy system using the 'smart tables' and selecting the ontology 'parent class' at https://metacyc.org/.

4. Nutrient analyses of seawater

Seawater samples were collected for nutrient analyses from the Niskin bottles at all 36 depth horizons. Dissolved inorganic nutrient (DIN) analyses were conducted at sea on a Bran + Luebbe AA3 HR segmented flow analyser by the CSIRO Hydrochemistry group, following standard spectrophotometric methods [12, 13, 14, 15, 16]. Detection limits for dissolved inorganic concentrations of silicate (Si) were 0.2 µmol L^{-1}, for phosphate (PO_4^{3-}) 0.01 µmol L^{-1}), for nitrate (NO_3^-) 0.01 µmol L^{-1}, for nitrite (NO_2^-) 0.015 µmol L^{-1}, and for ammonium (NH_4^+) 0.015 µmol L^{-1}.

5. Rate measurements

Stable isotope tracers (^{15}N and ^{13}C) were used to measure N_2 fixation rates, NO_3^- and NH_4^+ uptake rates and C assimilation rates (primary productivity). The rates have been published in Raes, van de Kamp [17] and are used in this study as a setting for the inferred functional metabolic pathways. Primary productivity and nitrogen assimilation data from this study are available at https://doi.pangaea.de/10.1594/PANGAEA.884052 (Raes et al., 2017) and https://doi.pangaea.de/10.1594/PANGAEA.885169 (Raes et al., 2018b).

6. Pigment analyses

Pigment analyses have been published in Raes, van de Kamp [17] and data are available at https://doi.pangaea.de/10.1594/PANGAEA.884052.

7. Statistical Analysis

The Ocean Data View (ODV; v.5.0.0.) software package was used to visualize the physical and bio-chemical data. The Phyloseq (v.1.28.0; McMurdie and Holmes [18]) and ggplot2 packages[19] were used to analyse, visualize and plot the microbiome data (including alpha; shown as Chao1 richness, and beta diversity) along the P15S GO-SHIP transect. The plyr package (v.1.8.4) was used to calculate means and summarise the data [20]. The ggpubr package [21] and Wilcoxon tests [22] were used to test for differences between ocean provinces and the Bonferoni method was used to control for multiple tests. Constrained Analysis of Principal Coordinates (CAP) plots were used to visualize the β-diversity variation between all four oceanographic provinces. Analysis of similarities (ANOSIM) was used to test whether we could identify statistical differences between the oceanographic provinces based on the sequence data and the MetaCyc pathway data. ANOSIM tests were performed on Bray-Curtis

dissimilarity matrices generated from square-root transformed data. The ‘indicspecies’ package (ver. 1.7.8; De Cáceres [23]) with the ‘multipatt’ function and 9999 permutations was used to identify indicator pathways in the four oceanographic provinces. Pearson’s phi coefficients of association [24] were calculated to determine significant indicator pathways in the four oceanographic provinces. The phi coefficient was corrected using the function=”r.g.” to accommodate for the fact some oceanographic provinces had more stations than others [25]. Twenty-two biotic and abiotic parameters were used to predict trends in the various metabolic pathways including primary productivity (PP) using boosted regression trees (BRT; Elith, Leathwick [26]). BRTs were implemented in the R v.3.6.1 software environment using the gbm.step function, a gaussian error structure, 10 fold cross-validation, and the following settings learning.rate=0.001, tree.complexit =10, bag.fraction=0.5 [26]. All models resulted in > 2050 trees. All statistical tests were conducted using the Vegan package version 2.5-6 [27] in R version 3.6.1 [28] and the PRIMER v7 software [29]. All code to reproduce the figures and analyses are available on https://github.com/EricRaes/marker_gene_manuscript

Supplementary Tables:

Supplementary Table 1: Shotgun metagenome statistics

Sample id	Depth (m)	utc_date sampled yyyymmdd	Latitude decimal degrees	Longitude decimal degrees	Sequencing depth after Merge and QC	Reads with hits to nr	Percent assigne d to nr	Percent assigned to KO
34369	10	4/05/2016	-66.3332	-170	92852539	16414249	17.7	22.9
34393	10	17/05/2016	-52.0028	-170.069	68368950	24869674	36.4	48.1
34402	10	19/05/2016	-47.4884	-169.984	1.09E+08	18020255	16.5	16.8
34415	10	29/05/2016	-41.7276	-173.951	87840501	23761652	27.1	29.7
34421	10	30/05/2016	-39.0728	-172.115	88607949	21924890	24.7	28.1
34430	10	2/06/2016	-35.0135	-169.991	68872839	24136787	35	38.2
34450	10	10/06/2016	-23.9956	-170.004	75714456	20174963	26.6	32.5
34458	10	12/06/2016	-19.9982	-170.002	60600664	25311787	41.8	35.2
34473	10	17/06/2016	-12.9974	-169.998	1.09E+08	23061283	21.2	24.4
34481	10	19/06/2016	-8.99877	-168.875	73863776	16801111	22.7	27.9
34499	10	24/06/2016	0.00383	-168.737	1.04E+08	21765018	20.9	24

Supplementary Table 2: ANOSIM results for OTU and MetaCyc pathways abundances.

Bacterial 16S rRNA OTU data

Ocean Provinces	R-value	Significance	Permutations
Southern Ocean and STF	0.823	0.001	999
Southern Ocean and SPSG	0.997	0.001	999
Southern Ocean and PED	1	0.001	999
STF and SPSG	0.859	0.001	999
STF and PED	0.999	0.001	999
SPSG and PED	0.448	0.001	999

MetaCyc data

Ocean Provinces	R-value	Significance	Permutations
Southern Ocean and STF	0.594	0.001	999
Southern Ocean and SPSG	0.988	0.001	999
Southern Ocean and PED	0.955	0.001	999
STF and SPSG	0.91	0.001	999
STF and PED	0.927	0.001	999
SPSG and PED	0.495	0.001	999

R values indicate the level of similarity between all sampled ocean provinces, with R=0 indicating strong similarity and R=1 indicative of a strong dissimilarity. Significant differences at the $p<0.001$ level.

Supplementary Figures

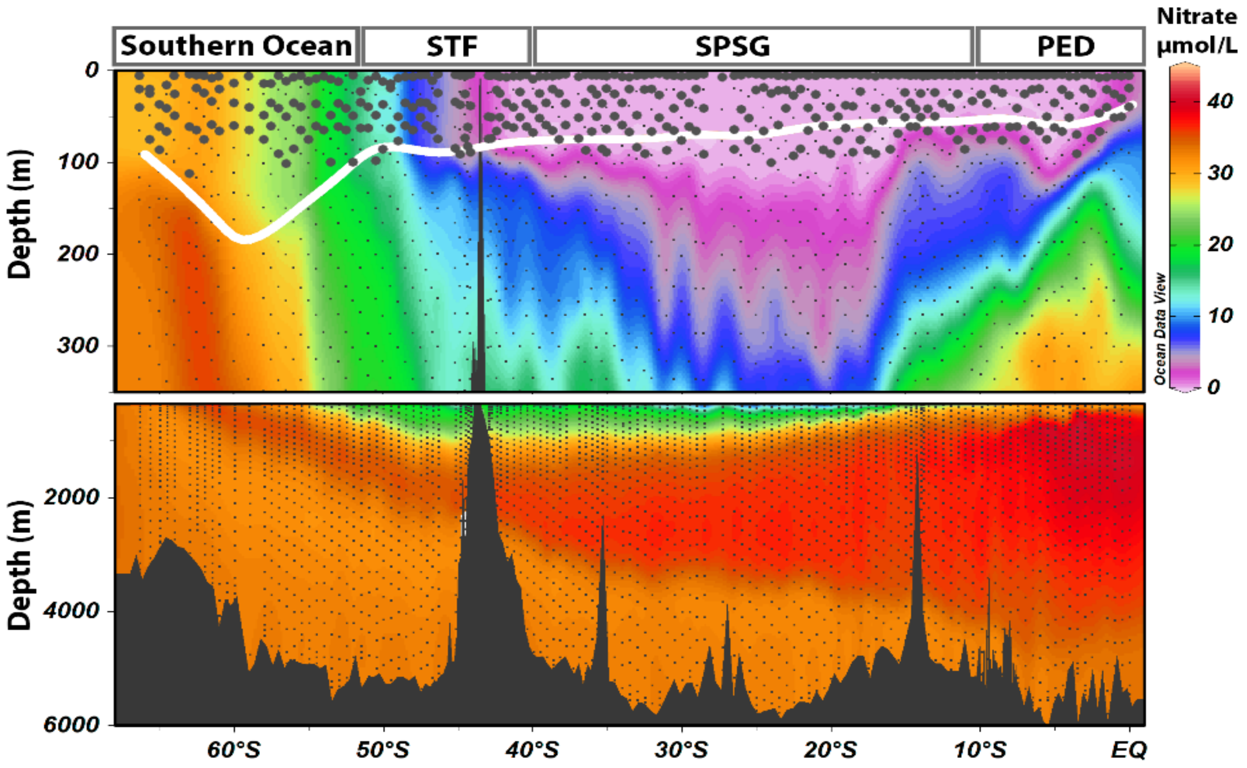

Supplementary Figure 1: Nitrate concentrations (µmol L^{-1}) along the P15S GO-SHIP transect from 66 °S to the equator. Top panel highlights data from the surface to 350m, and the bottom panel show nitrate concentrations from 350m to the bottom. The thick white line represents the MLD. The black circles on the top panel show where the DNA samples were collected and the little black dots on both panels shows denote the three sampling depths of 140 CTD stations. The depth range for Depth 1 was 6.6±4.1 m, for Depth 2 it was 35.3±13.1 m and for Depth 3, 70.7±18.0 m. The boxes on top of the figures mark the oceanographic provinces; the Southern Ocean, the Subtropical Frontal zone (STF), the South Pacific Subtropical Gyre (SPSG), and the Pacific Equatorial Divergence Province (PED).

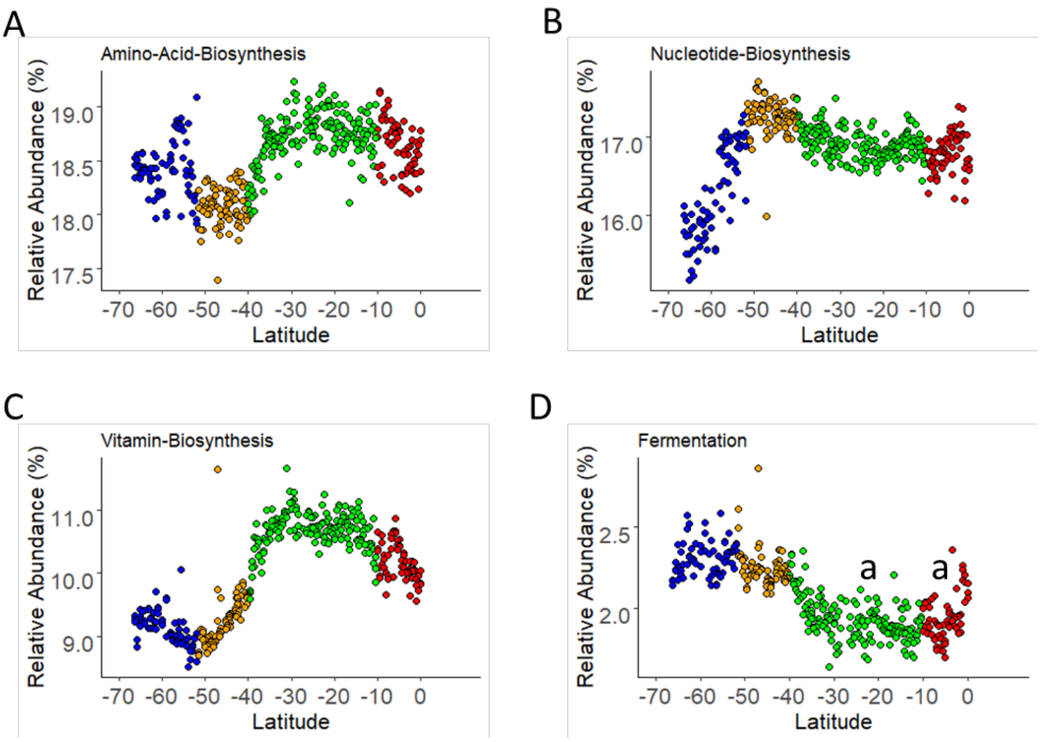

Supplementary Figure 2: Metabolic pathways. Significant differences were observed between provinces unless otherwise indicated by 'a'.

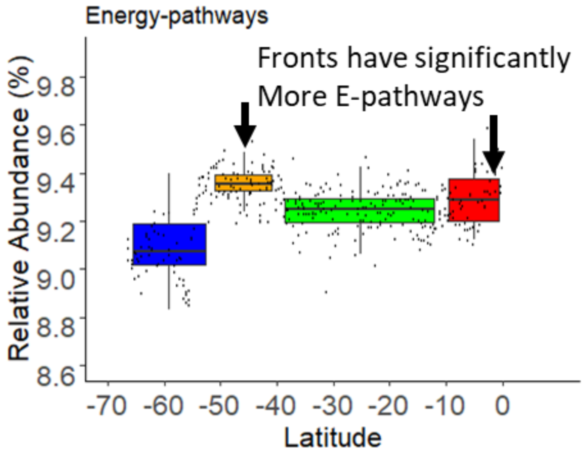

Supplementary Figure 3: Higher relative abundances for energy-pathways were found at frontal systems (STF and PED; highlighted by black arrows). The four oceanographic provinces described by Longhurst [30] are colour coded: the Southern Ocean (SO; blue sector;), the Subtropical Frontal zone (STF; orange sector), the South Pacific Subtropical Gyre Province (SPSG; green sector), the Pacific Equatorial Divergence Province (PED; red sector). Boxplots denote the medians and the interquartile ranges (IQR). Boxplots in show median values (centre line) is the median; the upper and lower quartiles are shown by box hinges; the

whiskers present 1.5x interquartile range and the points outside the whiskers are plotted individually.

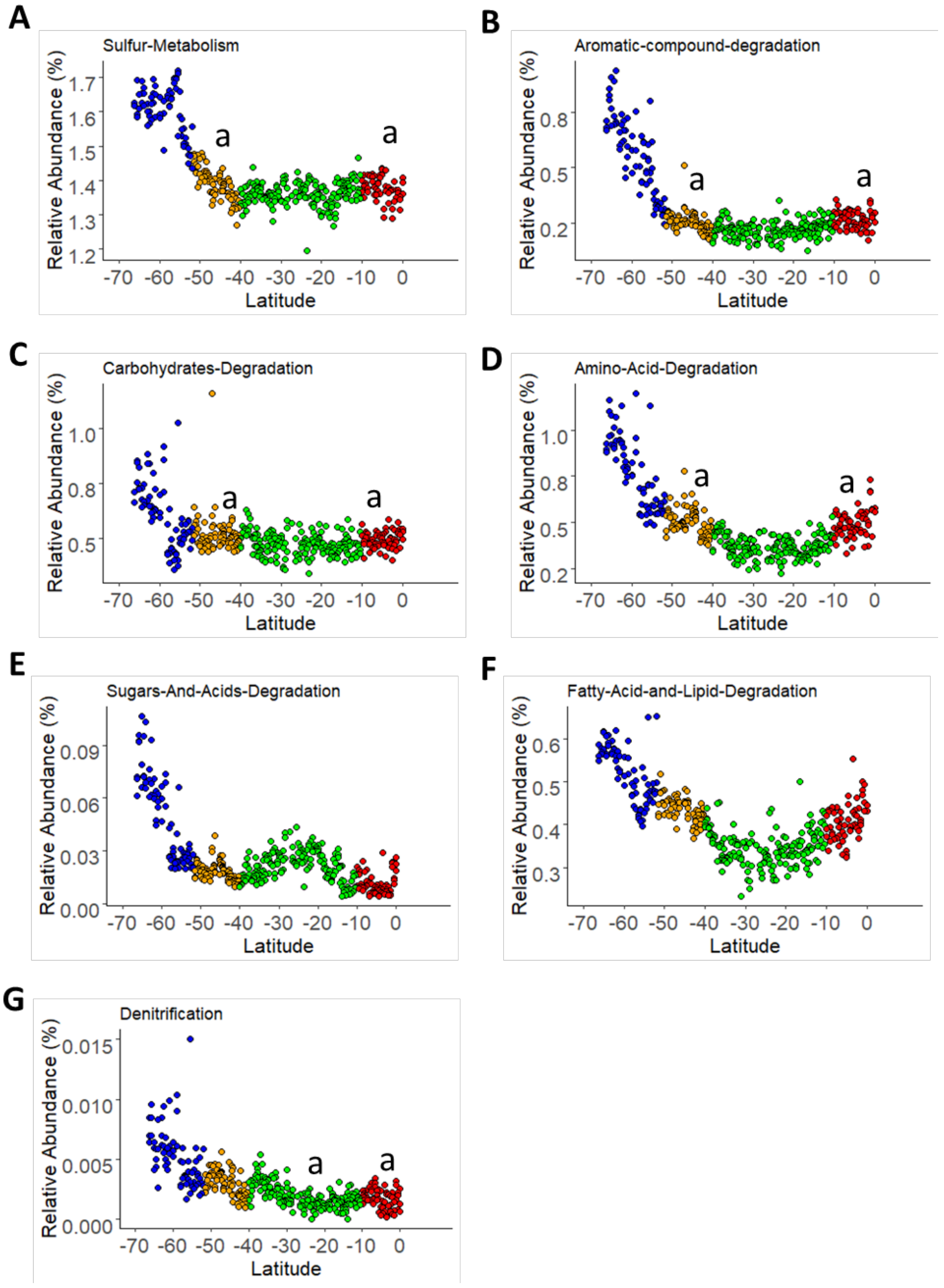

Supplementary Figure 4: Resulst from indicator analyses in the SO. The indicator analysis revealed a significant higher contribution of **a)** sulphu-metabolims, **b)** aromatic-compound degradation, **c)** carbodhydrate degradation, **d)** amonio-acid degradation, **e)** sugar and acid degradation, **f)** fatty acid and lipid degradation, **g)** denitrification pathways in the Southern Ocean. The oceanographic provinces are color-coded: the Southern Ocean (SO; blue), the Subtropical Frontal Zone (STF; orange), the South Pacific Subtropical Gyre Province (SPSG; green) and the Pacific Equatorial Divergence Province (PED; red). Significant differences were observed between provinces unless otherwise indicated by a.

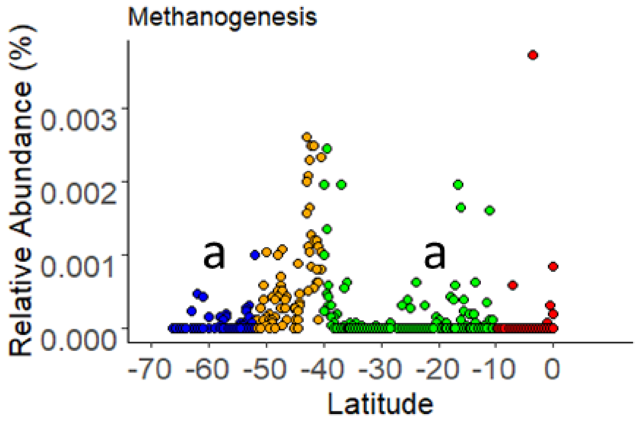

Supplementary Figure 5: Resulst from indicator analyses in the STF. The indicator analysis revealed a significant higher contribution of methanognesis pathways in the STF. The oceanographic provinces are color-coded: the Southern Ocean (SO; blue), the Subtropical Frontal Zone (STF; orange), the South Pacific Subtropical Gyre Province (SPSG; green) and the Pacific Equatorial Divergence Province (PED; red). Significant differences were observed between provinces unless otherwise indicated by a.

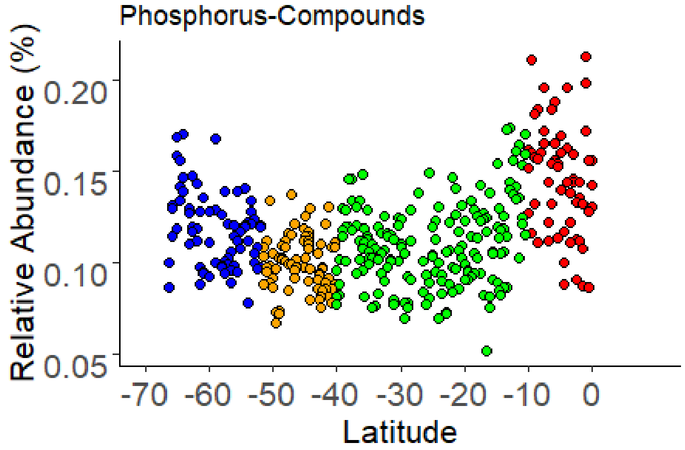

Supplementary Figure 6: Resulst from indicator analyses in the PED. The indicator analysis revealed a significant higher contribution of phoshorus-compound related pathways in the PED. The oceanographic provinces are color-coded: the Southern Ocean (SO; blue), the Subtropical Frontal Zone (STF; orange), the South Pacific Subtropical Gyre Province (SPSG; green) and the Pacific Equatorial Divergence Province (PED; red). Significant differences were observed between provinces unless otherwise indicated by a.

Supplementary references:

1. Bissett A*, et al.* Introducing BASE: the Biomes of Australian Soil Environments soil microbial diversity database. *GigaScience* **5**, 21 (2016).

2. Magoč T, Salzberg SL. FLASH: fast length adjustment of short reads to improve genome assemblies. *Bioinformatics* **27**, 2957-2963 (2011).

3. Edgar RC, Flyvbjerg H. Error filtering, pair assembly and error correction for next-generation sequencing reads. *Bioinformatics* **31**, 3476-3482 (2015).

4. Edgar RC. Search and clustering orders of magnitude faster than BLAST. *Bioinformatics* **26**, 2460-2461 (2010).

5. Andrews S. FastQC: a quality control tool for high throughput sequence data.). Babraham Bioinformatics, Babraham Institute, Cambridge, United Kingdom (2010).

6. Barbera P*, et al.* EPA-ng: Massively Parallel Evolutionary Placement of Genetic Sequences. *Systematic Biology* **68**, 365-369 (2018).

7. Czech L, Barbera P, Stamatakis A. Genesis and Gappa: processing, analyzing and visualizing phylogenetic (placement) data. *Bioinformatics*, (2020).

8. Douglas GM*, et al.* PICRUSt2 for prediction of metagenome functions. *Nat Biotechnol* **38**, 669-688 (2020).

9. Louca S, Doebeli M. Efficient comparative phylogenetics on large trees. *Bioinformatics* **34**, 1053-1055 (2017).

10. Ye Y, Doak TG. A Parsimony Approach to Biological Pathway Reconstruction/Inference for Genomes and Metagenomes. *PLOS Computational Biology* **5**, e1000465 (2009).

11. Markowitz VM*, et al.* IMG: the integrated microbial genomes database and comparative analysis system. *Nucleic acids research* **40**, D115-D122 (2012).

12. Aminot A, Kérouel R, Coverly SC. Nutrients in seawater using segmented flow analysis. *Practical guidelines for the analysis of seawater*, 143-178 (2009).

13. Armstrong F, Stearns C, Strickland J. The measurement of upwelling and subsequent biological process by means of the Technicon Autoanalyzer® and associated equipment. In: *Deep Sea Research and Oceanographic Abstracts*). Elsevier (1967).

14. Hydes D*, et al.* Determination of Dissolved Nutrients (N, P, SI) in Seawater With High Precision and Inter-Comparability Using Gas-Segmented Continuous Flow Analysers., (2010).

15. Kérouel R, Aminot A. Fluorometric determination of ammonia in sea and estuarine waters by direct segmented flow analysis. *Marine Chemistry* **57**, 265-275 (1997).

16. Grasshoff K, Kremling K, Ehrhardt M. *Methods of seawater analysis*. John Wiley & Sons (2009).

17. Raes EJ*, et al.* N2 Fixation and New Insights Into Nitrification From the Ice-Edge to the Equator in the South Pacific Ocean. *Frontiers in Marine Science* **7**, (2020).

18. McMurdie PJ, Holmes S. phyloseq: an R package for reproducible interactive analysis and graphics of microbiome census data. *PloS one* **8**, (2013).

19. Wickham H. ggplot2. *Wiley Interdisciplinary Reviews: Computational Statistics* **3**, 180-185 (2011).

20. Wickham H. The split-apply-combine strategy for data analysis. *Journal of Statistical Software* **40**, 1-29 (2011).

21. Kassambara A. ggpubr:"ggplot2" based publication ready plots. *R package version 01* **6**, (2017).

22. Wilcoxon F, Katti S, Wilcox RA. Critical values and probability levels for the Wilcoxon rank sum test and the Wilcoxon signed rank test. *Selected tables in mathematical statistics* **1**, 171-259 (1970).

23. De Cáceres M. How to use the indicspecies package (ver. 1.7. 1). *R Proj* **29**, (2013).

24. Chytrý M, Tichý L, Holt J, Botta-Dukát Z. Determination of diagnostic species with statistical fidelity measures. *Journal of Vegetation science* **13**, 79-90 (2002).

25. Tichy L, Chytry M. Statistical determination of diagnostic species for site groups of unequal size. *Journal of Vegetation Science* **17**, 809-818 (2006).

26. Elith J, Leathwick JR, Hastie T. A working guide to boosted regression trees. *Journal of Animal Ecology* **77**, 802-813 (2008).

27. Oksanen J*, et al.* The vegan package. *Community ecology package* **10**, 631-637 (2007).

28. Team RC. R: A language and environment for statistical computing. (2013).

29. Clarke K, Gorley R. Primer. *PRIMER-e, Plymouth*, (2006).

30. Longhurst AR. *Ecological geography of the sea*. Elsevier (2010).
