# Supplementary material for: Metabolic pathways inferred from a bacterial marker gene illuminate ecological changes across South Pacific frontal boundaries
Source: Nat Commun. 2021 Apr 13;12:2213. doi: 10.1038/s41467-021-22409-4 (PMC8044245; doi:10.1038/s41467-021-22409-4)
Supplement: Supplementary file 3 — Description of Additional Supplementary Files [file 41467_2021_22409_MOESM3_ESM.docx]

Supplementary Data

**Metabolic pathways inferred from a bacterial marker gene illuminate ecological changes across South Pacific frontal boundaries.**

**Eric J. Raes^1*^, Kristen Karsh^1^, Swan L. S. Sow^1,2,3^, Martin Ostrowski^4^, Mark Brown^5^, Jodie van de Kamp^1^, Rita M. Franco-Santos^2^, Levente Bodrossy^1^, Anya M. Waite^6^**

1) CSIRO Oceans and Atmosphere, GPO Box 1538, Hobart, TAS, 7004 Australia

2) Institute for Marine and Antarctic Studies, University of Tasmania, 20 Castray Esplanade, Hobart 7004, TAS, Australia

3) NIOZ Royal Netherlands Institute for Sea Research, Department of Marine Microbiology and Biogeochemistry, P.O. Box 59, 1790 AB Den Burg, The Netherlands

4) Climate Change Cluster, University of Technology Sydney, Sydney, NSW, Australia
5) School of Environmental and Life Sciences, The University of Newcastle, Callaghan, NSW, Australia
6) Ocean Frontier Institute and Dept. of Oceanography, Dalhousie University, Halifax, B3H 4R2 Nova Scotia, Canada

*** Corresponding author:**Eric Jorden Raes
[ejraes@gmail.com](mailto:ejraes@gmail.com)

**Supplementary Data 1:** The 400 inferred MetaCyc pathways from the PICRUSt2 analysis. MetaCyc pathways are collapsed into 41 secondary superclasses and ontology parents of MetaCyc pathways are also shown at two levels. Attached as a separate Supplementary Data 1 file in .csv format.

**Supplementary Data 2:** BRT model outputs from 75 observations and 22 predictors. See tables for the relative influence (%) for the 22 biotic and abiotic predictors. Attached as a separate Supplementary Data 2 file in .csv format.

**Supplementary Data 3:** Details the relative abundance of each sequence at the 97% similarity threshold with an NSTI >2. Attached as a separate Supplementary Data 3 file in .csv format.
